# A cost-effectiveness analysis of capecitabine maintenance therapy versus routine follow-up for early-stage triple-negative breast cancer patients after standard treatment from a perspective of Chinese society

**DOI:** 10.1186/s12916-022-02516-9

**Published:** 2022-09-26

**Authors:** Ji-Bin Li, Zhuo-Chen Lin, Martin C. S. Wong, Harry H. X. Wang, Mengmeng Li, Su Li

**Affiliations:** 1 Department of Clinical Research, Sun Yat-Sen University Cancer Center, Guangzhou, People’s Republic of China; 2 State Key Laboratory of Oncology in South China, Collaborative Innovation Center for Cancer Medicine, Guangzhou, People’s Republic of China; 3 Department of Medical Records, The First Affiliated Hospital, Sun Yat-Sen University, Guangzhou, People’s Republic of China; 4 The Jockey Club School of Public Health and Primary Care, Faculty of Medicine, The Chinese University of Hong Kong, Hong Kong SAR, People’s Republic of China; 5 Chinese Academy of Medical Sciences and Peking Union Medical College, Beijing, People’s Republic of China; 6 School of Public Health, The Peking University, Beijing, People’s Republic of China; 7 School of Public Health, Sun Yat-Sen University, Guangzhou, People’s Republic of China; 8 Department of Cancer Prevention Research, Sun Yat-Sen University Cancer Center, Guangzhou, People’s Republic of China

**Keywords:** Cost-effectiveness, Quality-adjusted life years, Capecitabine maintenance therapy, Early-stage triple-negative breast cancer

## Abstract

**Background:**

Capecitabine maintenance therapy is safe and efficacious for early-stage triple-negative breast cancer (TNBC) patients, but the cost-effectiveness of its long-term use has not been investigated. Here, we evaluated the cost-effectiveness of capecitabine maintenance therapy, compared with routine follow-up, in early-stage TNBC patients after standard treatment from a perspective of Chinese society.

**Methods:**

A three-state Markov model based on the data from the SYSUCC-001 trial was constructed to estimate the cost-effectiveness of capecitabine maintenance therapy in a month cycle over a period of 30-year time horizon. A 5% annual discount rate was set for all costs and benefits. One-way and probabilistic sensitivity analyses were performed to explore the model uncertainties. The main outcomes include quality-adjusted life years (QALYs), incremental cost-effectiveness ratio (ICER), and the number needed to treat (NNT) to prevent one additional event.

**Results:**

Compared with routine follow-up, 1-year capecitabine maintenance therapy yielded an additional 1.29 quality-adjusted life years (QALYs) at an additional cost of $3391.70, with an ICER of $2630.53 (95% CI: $1159.81–$5090.12) per QALY gained. The ICER was considerably lower than the recommended willingness-to-pay (WTP) threshold (i.e., $28,130.00 per QALY). The results were sensitive to the discount rate, drug cost, and treatment cost after relapse. Further, the NNT to prevent one additional relapse case was 29.2 (95% CI: 13.2–196.6), 16.7 (95% CI: 8.4–111.6), and 12.0 (95% CI: 5.7–82.6) at 1, 2, and 5 years, respectively.

**Conclusions:**

One-year capecitabine maintenance therapy for early-stage TNBC after standard treatment, compared with routine follow-up, was found to be highly cost-effective with promising clinical benefits and acceptable increased costs. Real-world studies are warranted to validate our findings in the future.

**Supplementary Information:**

The online version contains supplementary material available at 10.1186/s12916-022-02516-9.

## Background

Breast cancer has become the most frequently diagnosed cancer worldwide in 2020, with an estimated 2.3 million new cases globally [1]. A rapid increase in the incidence and burden of breast cancer has also been observed in China [2, 3]. Currently, the medical costs of treating breast cancer remain a considerable economic burden for the Chinese medical insurance and healthcare system, and decision-making based on cost-effectiveness analysis is warranted.

Triple-negative breast cancer (TNBC), characterized by negative expression of estrogen, progesterone, and human epidermal growth factor receptor-2 [4], is the most aggressive breast malignancy, accounting for 19% of all types of breast cancer in Chinese patients [5]. TNBC is not sensitive to endocrine therapy or molecular targeted therapy. Compared with other types of breast cancer, the treatment options of TNBC are still limited, with high recurrence and metastasis rates and poor prognosis [6, 7]. Adjuvant chemotherapy remains the main choice for early-stage TNBC patients, but the efficacy is poor [8, 9].

Two recent meta-analyses have shown the clinical benefits of adding adjuvant capecitabine concurrently with or sequentially after standard chemotherapy treatment in improving the prognosis of early-stage TNBC patients [10, 11]. However, the efficacy was dependent on the duration of adjuvant capecitabine treatment, and the substantial improvement of disease-free survival (DFS) was only observed in three trials with adjuvant capecitabine for ≥ 6 cycles, including CREATE-X with 6–8 cycles [12], CIBOMA/2004 with 8 cycles [13], and SYSUCC-01 with 1-year maintenance [14]. Thus, the SYSCC-001 represents the multicenter trial with the longest duration of adjuvant capecitabine treatment [14]. The SYSUCC-001 trial evaluated the efficacy and safety of 1-year low-dose capecitabine maintenance therapy compared with routine follow-up recommended by clinical guidance as a control group in early-stage TNBC patients after standard treatment [15]. The trial showed a statistically significant improvement in 5-year DFS by 9.8% in the capecitabine maintenance group compared with the observational group (routine follow-up). However, the improvement in 5-year overall survival (85.5% vs. 81.3%) and 5-year locoregional recurrence-free survival (85.0% vs. 80.8%) between the groups were not significantly different, which was similar to two previous trials [12, 13]. Further, it is observed that capecitabine maintenance therapy lasting for 1 year after standard treatment is associated with increased costs and additional toxicity.

Despite the evidence of potential efficacy in reducing relapse rate from capecitabine maintenance therapy in the adjuvant setting, its cost-effectiveness remains unclear but is necessary for policy-makers and clinical practice. In this study, we conducted a cost-effectiveness analysis to evaluate the costs and clinical benefits of 1-year capecitabine maintenance therapy for early-stage TNBC patients from the perspective of Chinese society.

## Results

### Base-case model

Patients in the capecitabine maintenance group yielded 9.30 QALYs compared with 8.01 QALYs for patients in the observational group. The total cost was $9106.67 in the capecitabine maintenance group and $5714.96 in the observational group, respectively. The capecitabine maintenance therapy provided an additional 1.29 QALYs at an additional cost of $3391.70. The ICER was $2630.53 (95% CI: $1159.81–$5090.12) per QALY gained, which was considerably lower than the recommended WTP threshold (i.e., $28,130 per QALY), indicating that 1-year capecitabine maintenance therapy as adjuvant treatment was highly cost-effective for early-stage TNBC patients after standard treatment, compared to routine follow-up. A sensitivity analysis was conducted to test the uncertainty of ICER according to alternative survival functions (i.e., Exponential, Weibull, Log-logistic, and Gamma). The results showed that the ICERs were consistently lower than the recommended WTP with a range from $2176.91 per QALY gained on Gamma distribution to $2876.78 per QALY gained on exponential distribution, indicating the robustness of ICER results on survival functions (Additional file 1: Table S5).

### Sensitivity analyses

**Fig. 1:**
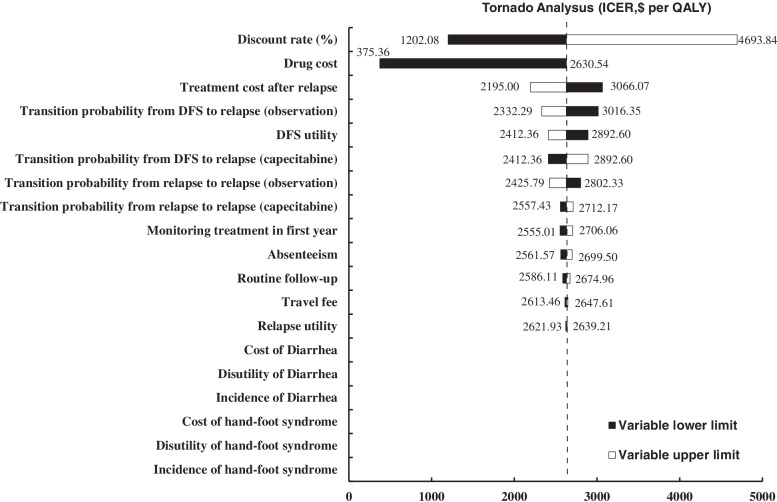
Tornado diagram of one-way sensitivity analysis. DFS, disease-free survival; ICER, incremental cost-effectiveness ratio; QALY, quality-adjusted life year

Results of the one-way sensitivity analysis are presented in the tornado diagram (Fig. 1). The parameters that considerably impacted ICER estimates included the annual discount rate, monthly drug cost, and treatment cost after relapse. Within the range of each parameter specified in Table 2, the ICER was consistently below the recommended WTP threshold.

**Fig. 2:**
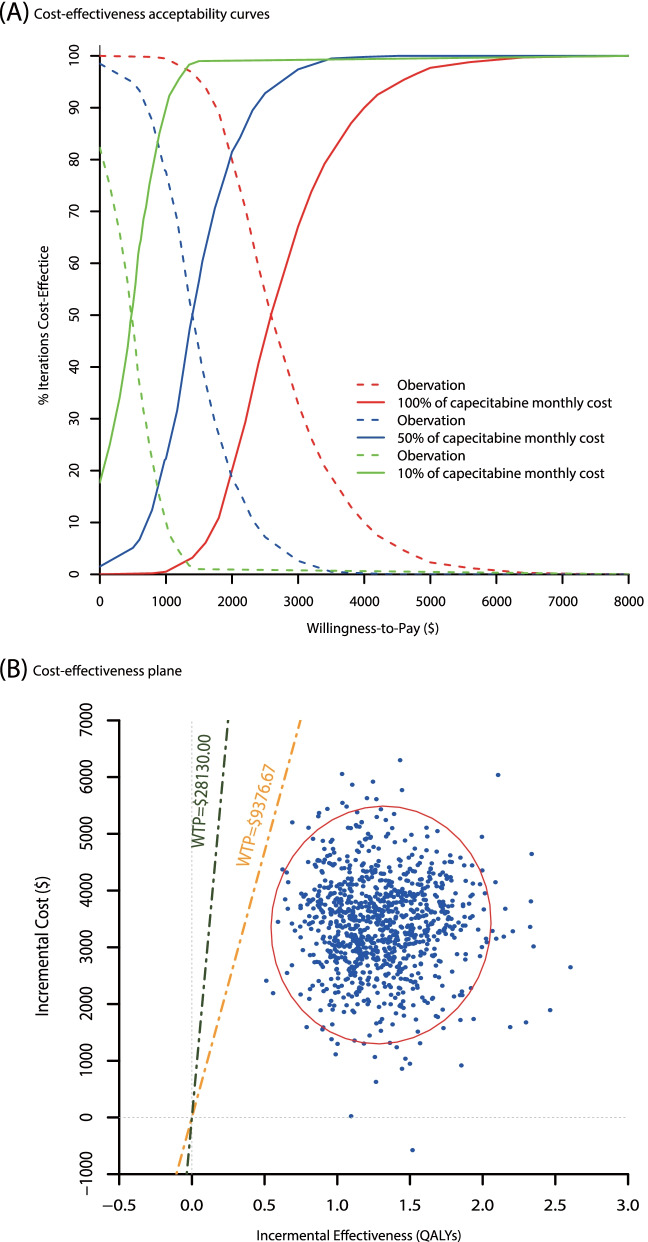
Probabilistic sensitivity analyses for capecitabine maintenance therapy. **A** Cost-effectiveness acceptability curves at different discounts of monthly capecitabine cost. **B** Cost-effectiveness plane

The cost-effectiveness acceptability curve indicated that 1-year capecitabine maintenance therapy was more likely to be cost-effective compared with routine follow-up when the WTP threshold was above $2610.00 per QALY gained (Fig. 2). The probability that capecitabine maintenance therapy was cost-effective was 100%, indicating that capecitabine maintenance therapy was dominantly cost-effective, compared with routine follow-up, in improving the prognosis of early-stage TNBC patients after standard treatment. If the monthly capecitabine cost was 50% and 10% in the base-case model, the WTP threshold that capecitabine maintenance therapy could be treated as cost-effective was decreased to $1406.2 and $482.7 per QALY gain, respectively (Fig. 2A).

### Number needed to treat

**Fig. 3:**
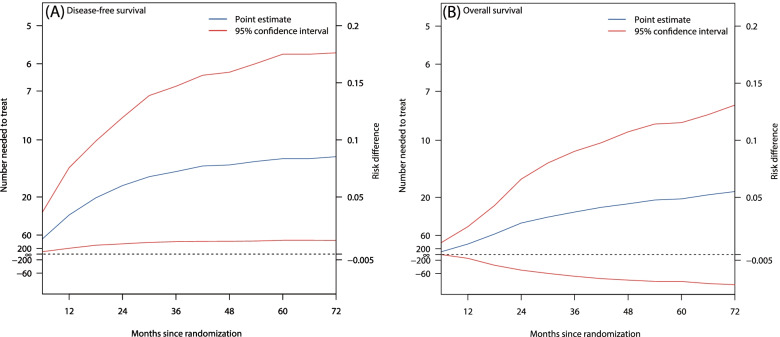
Risk difference and number needed to treat for (**A**) disease-free survival and (**B**) overall survival. Blue lines represented point estimates and red lines represented 95% confidence intervals estimated using the bootstrapping method

The absolute risk reduction of relapse rate at the 1, 2, and 5 years since the date of the randomization in the capecitabine maintenance group, compared with routine follow-up, were 3.4%, 6.0%, and 8.4%, and the NNTs to prevent one additional relapse event was 29.2 (95% CI: 13.2–196.6), 16.7 (95% CI: 8.4–111.6), and 12.0 (95% CI: 5.7–82.6), respectively (Fig. 3A). The additional costs of capecitabine maintenance therapy, compared with routine follow-up, to prevent one additional relapse were $138,361.75, $79,131.55, and $56,860.99 at 1, 2, and 5 years, respectively. However, compared with routine follow-up, the NNT to prevent one additional death of capecitabine maintenance therapy was not statistically significant (Fig. 3B).

## Discussion

Substantial survival benefits of adjuvant capecitabine therapy for early-stage TNBC have been demonstrated in previous literature [10, 14, 16]. The evidence of its cost-effectiveness is a pivotal consideration for treatment decision-making in developing countries, especially for those with limited health resources. To the best of our knowledge, this is the first study to evaluate the economic outcomes of adjuvant capecitabine therapy for early-stage TNBC patients after they complete standard treatment based on the latest clinical evidence from a multicenter, randomized controlled trial in China.

Our findings showed that 1-year capecitabine maintenance therapy was highly cost-effective as compared to the currently recommended routine follow-up among early-stage TNBC patients. The ICER of $2630.53 per QALY gained was considerably lower than the WTP threshold from a Chinese societal perspective. One-way sensitivity analysis consistently demonstrated the robustness of cost-effectiveness results in the model’s uncertainty. The PSA revealed that capecitabine maintenance therapy was a dominated option under the WTP threshold. In addition, the results from NNTs further confirmed the clinical benefits of adjuvant capecitabine therapy in preventing relapse of early-stage TNBC patients.

The tornado diagram revealed that capecitabine cost was a substantially influential parameter for the robustness of the model. However, the ICERs were consistently lower than the WTP threshold at a varying parameter range, with ICER ranging from $375.36 to $2630.54 per QALY gained. Drug price has a considerable impact on ICER in China [17]. If the unit price of capecitabine is reduced to 50% and 10% of the base-case value, capecitabine maintenance therapy could be cost-effective with a WTP of $1406.2 and $482.7 per QALY gained, respectively. Therefore, it could be beneficial to further reduce the ICER by decreasing the marketing price of capecitabine.

Individuals’ WTP plays an important role in the Chinese healthcare system [18]. Currently, there is no unanimously agreed cost-effectiveness threshold in China. Our results suggest that capecitabine maintenance therapy is highly cost-effective if the WTP is above $2610.00 per QALY gained. The WHO suggested that an intervention could be considered highly cost-effective when its ICER is less than per-capita GDP, cost-effective when its ICER is between one to three times per-capita GDP, and not cost-effective when its ICER is over three times per-capita GDP [19, 20]. In our study, the WTP threshold was set as three times the per-capita GDP of China (i.e., $28,130). There is a huge gap in per-capita GDPs across different regions of mainland China—from the lowest of $5566.4 in Gansu province to the highest of $25,499.0 in the Beijing municipality in 2020 (with an exchange rate of $1 = ¥6.4665) [21]. However, the ICER of capecitabine maintenance therapy is consistently less than per-capita GDP even in the least developed region of China, suggesting that the added cost of capecitabine maintenance therapy could be entirely cost-effective for general Chinese breast cancer patients.

The per-capita GDP and WTP thresholds vary widely worldwide [22, 23]. Our results suggest that adjunct capecitabine maintenance therapy could be treated as cost-effective when the per-capita GDP is above $876.7 (i.e., one-third of ICER), while only 24 out of 190 countries/regions (12.6%) are with per-capita GDP less than $876.7 [23]. Although the present study focused on Chinese society, given the relatively low ICER values in our study, we believe that the findings might have reference values for policy-making of using adjunct capecitabine maintenance therapy worldwide, in the developed countries with higher WTP thresholds (e.g., the USA) [24] and in other developing countries with relatively lower WTP thresholds (e.g., South Africa) [25].

NNT is an effective index to express results in a clinically meaningful way. Our results showed that, to prevent one relapse event over a 2-year and 5-year period, 16.7 and 12.0 TNBC patients would have to be treated with adjuvant capecitabine, respectively. The NNT of DFS at 5-year in our study was slightly higher than that in the CREATE-X trial [12], which was 7.3 of adjuvant capecitabine versus observation among HER2-negative residual invasive breast cancer after neoadjuvant chemotherapy, but lower than that in GEICAM-CIBOMA trial [13], which was 35.7 of capecitabine versus observation among operable triple-negative breast cancers. Apart from the discrepancies concerning the study design, population, and adjuvant treatment strategies, the differences might be partially attributed to the lower rate of neoadjuvant chemotherapy in the SYSUCC-001 trial (i.e., only 5.8%) [14] and the short duration of capecitabine therapy (i.e., 24 weeks) in GEICAM-CIBOMA trial [13]. However, our findings revealed non-added clinical benefits of capecitabine maintenance compared with routine follow-up for NNT to prevent one additional death, which was consistent with the results of the SYSUCC-001 trial whereby OS was not significantly improved by capecitabine maintenance therapy [14]. The reason might be that survival after relapse was multifactorial, consisting of contributions from clinical factors and subsequent treatment modalities (e.g., chemotherapy, immunotherapy, targeted therapy, or their combination) apart from capecitabine therapy [26].

The major advantage of this study was that the findings were based upon a recent phase III, multicenter, randomized clinical trial in China, and the survival probabilities over the study period were extrapolated using individual-level data from the SYSUCC-001 trial, rather than extracting the survival probability from published Kaplan–Meier curves by digitizer software [27]. In addition, the 30-year time horizon used in this study could capture the long-term impacts of capecitabine maintenance therapy for early-stage TNBC patients after standard treatment, and the findings could provide lifetime evidence both for clinical practice of selecting long-term maintenance treatment and Chinese medical insurance policy-making.

However, the findings should be interpreted cautiously due to the following limitations. First, information from clinical trials might not fully represent real-world clinical consultations, considering that patients who did not fulfill the eligibility criteria for clinical trials were excluded. Real-world studies are warranted to validate the findings in our study. Second, because data were missing on quality of life from the SYSUCC-001 trial, the utilities for the state of DFS and relapse and the disutility for severe adverse events were extracted from previous reports. However, the model outcomes were robust when varying the utility/disutility values in the sensitivity analyses. Third, it is assumed that all patients in the interventional group adhered to 1-year capecitabine maintenance therapy without interruption, representing an idealized scenario and a source of uncertainty. However, the SYSUCC-001 trial reported a high completion rate of 1-year therapy (82.8%) and a very low rate of treatment interruption (4.1%) due to unacceptable toxicity (i.e., hand-foot syndrome). Furthermore, the impact of treatment discontinuation was uncertain and difficult to estimate. Based on the considerations, the influence of treatment interruption on cost-effectiveness was not considered in the proposed model, which might be a source of uncertainty for the results. Fourth, this study did not consider other potential direct non-medical costs except for travel costs. However, as capecitabine was orally administered, the impact of other direct non-medical costs seemed small. Fifth, the cost-effectiveness estimates at different durations of low-dose capecitabine maintenance therapy were unclear and require further exploration, considering that relapse mainly occurred within the first 2 to 3 years since diagnosis [28, 29]. Fifth, the international generalizability of our findings should be cautious, given that costs and WTP might vary substantially across different regions/countries. Although the medical costs based on the price charged at the Sun Yat-sen University Cancer Center could represent circumstances of the current standard treatment situation in China, the potential variation of medical costs across regions in China should be considered. Sixth, some cost parameters (i.e., cost of managing severe hand-foot syndrome and treatment after relapse) were estimated based on a small sample or consultation with oncologists from Sun Yat-sen University Cancer Center rather than applying the Delphi panel method, which might be a source of bias. In addition, a few studies have evaluated the cost-effectiveness of capecitabine in combination with other agents (e.g., docetaxel, ixabepilone, lapatinib) among advanced/metastatic breast cancer [30–33]. Their cost-effectiveness merits further exploration among early-stage breast cancer patients.

## Conclusions

Capecitabine maintenance therapy was found to be highly cost-effective compared with routine follow-up in reducing the relapse risk of early-stage TNBC patients after standard treatment from the perspective of Chinese society. The findings could be helpful to guide clinicians in making an optimal decision for treating early-stage TNBC patients and be useful for medical policy-making in China. Further evidence from real-world studies is warranted to validate the efficacy of long-term survival and its safety, as well as the health economics of this therapy in early-stage TNBC patients after standard treatment.

## Methods

### Trial background

The target population of the present study was from the SYSUCC-001 trial conducted in 13 academic centers and clinical sites in mainland China [14]. Briefly, eligible patients were women who had pathologically confirmed invasive breast ductal carcinoma, were hormone receptor and ERBB2 negative, were early stage with T1b-3N0-3cM0, and completed standard adjuvant chemotherapy. Eligible patients were randomly assigned (1:1) into either the capecitabine maintenance (interventional) group or the observational (control) group within 4 weeks after completion of standard adjuvant chemotherapy. In the observational group, patients were routinely followed up according to the clinical guidance. In the capecitabine maintenance group, patients received oral capecitabine at 650 mg/m^2^ twice daily continuously for 1 year without interruption. Capecitabine was given to patients via monthly prescriptions at the hospital. Patients in the capecitabine maintenance group had their blood, liver, and renal function tests taken monthly to monitor the safety of the medication. In both groups, patients were followed up by physical examination, assessment of menopausal status, breast ultrasound, and abdominal ultrasound by trained oncologists every 3 months during years 1 to 2, every 6 months during years 3 to 5, and yearly thereafter. Mammography and chest x-ray were performed yearly in both groups.

### Clinical data

In SYSUCC-001 [14], a total of 434 patients (median age: 46, range: 24–70) were included in the analysis, including 221 in the capecitabine maintenance group and 213 in the observational group; the median age was 45 for the capecitabine maintenance group and 48 for the observational group. After a median follow-up of 61 months, there were 93 recurrence events and 72 death events. The 5-year DFS was 82.8% in the capecitabine group vs. 73.0% in the observational group, and the 5-year OS was 85.5% in the capecitabine group vs. 81.3% in the observational group, respectively.

### Model structure

**Fig. 4:**
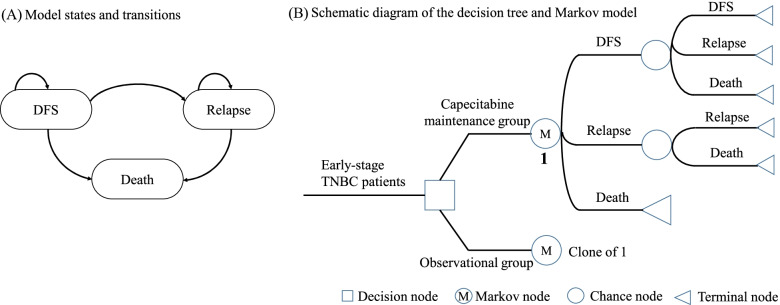
Markov model structure. **A** Model states and transitions. **B** Schematic of decision tree and Markov model. DFS, disease-free survival; TNBC, triple-negative breast cancer

A Markov state-transition model, using the TreeAge Pro 2005 software (TreeAge Software Inc, Williamstown, MA), was developed to evaluate the costs and effectiveness of 1-year capecitabine maintenance therapy for early-stage TNBC patients who had completed standard adjuvant chemotherapy compared with routine follow-up from the perspective of Chinese society. The three mutually exclusive health states of DFS, relapse, and death were included in the state-transition model (Fig. 4A). The abbreviated decision tree and Markov model are presented in Fig. 4B. All simulated patients started from the DFS state and could stay in the DFS state or move to the relapse or death state at the next cycle length due to corresponding transition probabilities; patients who progressed to the relapse state could only stay in the relapse state or move to the death state. Death was referred to as the absorbing state. The Markov model parameters were collected from the SYSUCC-001 trial and literature. Considering that the age peak of breast cancer incidence was around 45–49 years in China [34], and the median age of the capecitabine maintenance group was 45 in the SYSUCC-001 trial [14], the simulation was conducted from the age of 45 in a monthly cycle. The 1-month cycle length was used in line with the drug schedule. A 30-year time horizon was selected to capture the lifetime impacts of capecitabine maintenance therapy for early-stage TNBC patients [35]. During the 30-year period, the majority of patients would die. Transition probabilities were estimated based on individual-level data from the SYSUCC-001 trial. The study complied with the Consolidated Health Economic Evaluation Reporting Standards 2022 (CHEERS 2022) statement (Additional file 1: Table S1) [36].

### Transition probability

**Fig. 5:**
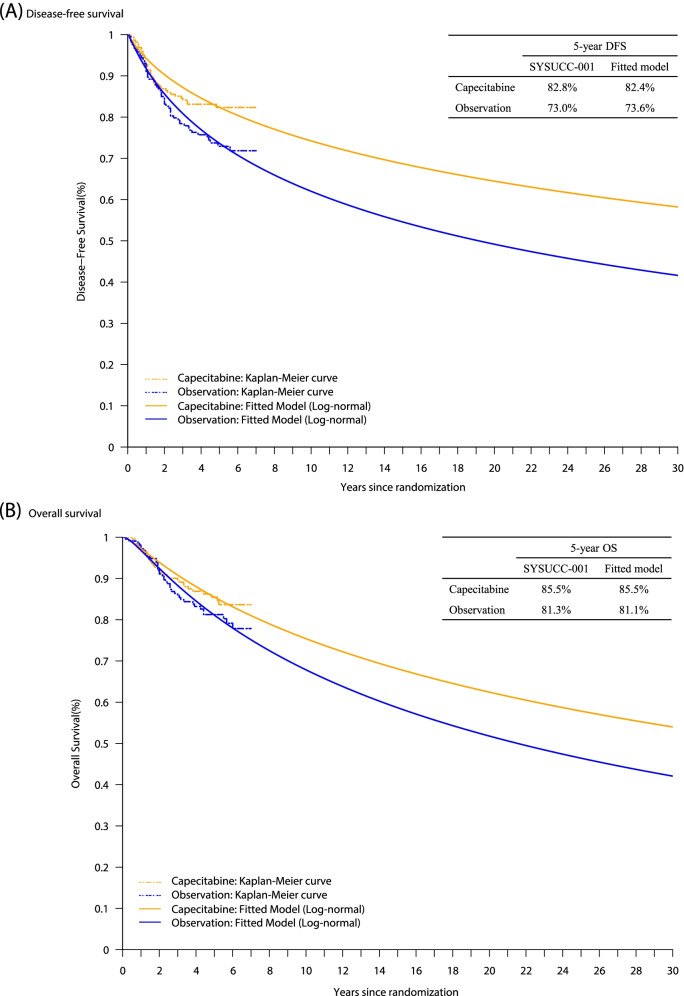
The fitted survival curves of log-normal distribution and original Kaplan–Meier curves for capecitabine maintenance group and observational group. **A** Disease-free survival. **B** Overall survival. DFS, disease-free survival; OS, overall survival

The time horizon in the Markov model is beyond the period of the SYSUCC-001 trial. It is generally preferred to apply a fitted parametric distribution over using raw survival data in cost-effectiveness studies when the simulated time horizon is much longer than the trial. To optimally extrapolate the survival probabilities over a 30-year time horizon, five common parametric distributions with Exponential, Weibull, Log-normal, Log-logistic, and Gamma were fit to the original individual-level data from the SYSUCC-001 trial (Additional file 1: Table S2). The selection of optimal parametric distribution for survival function was based on the recommended criteria [37, 38], including the lower values of Akaike information criterion (AIC), Bayesian information criterion (BIC), absolute of –2log-likelihood, and the sum of the squared errors (SSE) of predictions of survival curve over the observed time span, along with the visual comparison of modeled against Kaplan–Meier survival curves. The goodness-of-fit results indicated that log-normal distribution had a better fit for individual-level data of both DFS and OS (Additional file 1: Table S3, and Figure S1**)** and was finally selected for the cost-effectiveness analysis. The internal validation of the Log-normal distribution was confirmed by the agreement of modeled clinical outcomes with trial data in terms of the 5-year DFS and OS (Fig. 5). The estimated parameters of Log-normal distribution in Table 1 were applied to fit the 30-year time horizon survival curves of the two groups (Fig. 5). Time-dependent state-transition probabilities in each cycle were calculated using the formula $P\left(t\to 1\right)=1\frac{S\left(t+1\right)}{S\left(t\right)}$ at a given cycle *t* in the Markov model, where $S\left(t\right)=1-\Phi \left[\frac{\mathrm{ln}t-\mu }{\sigma }\right]$ represents the survival probability at time *t* using parameters (*μ*, *σ*) of Log-normal in Table 1 [37, 39, 40]. The transition probability from DFS to death was set as the age-specific natural mortality rate of the general Chinese women population in 2020 [41], according to previous literature [42, 43]. A time-dependent transition probability matrix sample is presented in Table S4 (Additional file 1).

**Table 1:** The input parameters of the log-normal survival model

	Best fitting model	μ	σ
Disease-free survival			
Capecitabine	Log-normal	6.400	0.908
Observation	Log-normal	5.437	0.753
Overall survival			
Capecitabine	Log-normal	6.074	0.627
Observation	Log-normal	5.555	0.505

### Cost estimates

Costs were calculated from the perspective of Chinese society. Direct medical costs included capecitabine, imaging, and laboratory tests for monitoring treatment safety and routine follow-up, managing severe adverse events (grade 3/4), and subsequent treatment after relapse. Since capecitabine (500 mg/piece) was only obtained by overseas importation to China at the beginning of the SYSUCC-001 trial (2010) and its generic was available in China at the end of 2013; Thus, its cost in the base-case model was therefore estimated on the imported unit price. Capecitabine has now entered the centralized drug procurement list in China; its price range was obtained from the Chinese Drug Bidding Database [44]. The unit costs of imaging, laboratory tests, and routine follow-up were based on the price charged at the Sun Yat-sen University Cancer Center (Guangzhou, China) in 2020. As the grade 1/2 adverse events were manageable with standard monitoring, we only involved the costs of grade 3 or above adverse events related to capecitabine maintenance therapy with a significant group difference in incidence rate, including hand-foot syndrome and diarrhea [11]. The incidence rates of severe adverse events were estimated from the SYSUCC-001 [14] and two relevant RCTs [12, 13]. The costs of managing severe adverse events were derived from the Sun Yat-sen University Cancer Center (for hand-foot syndrome) and literature (for diarrhea) [45]. For relapsed patients, chemotherapy, targeted therapy, immunotherapy, or surgery were given at the discretion of the oncologists. The average monthly treatment cost after relapse was estimated for individual total treatment cost by dividing his/her survival months after relapse, based on 16 dead cases (9 patients in the observation group and 7 patients in the capecitabine maintenance group) that occurred in Sun Yat-sen University Cancer Center. The reasonability of the estimated monthly treatment cost after relapse was confirmed by consulting an oncologist. Given that capecitabine was orally administered, we only involved the travel fee for drug collection and/or routine follow-up. Other direct non-medical costs were not considered in this study. The travel fee was set as $12.37 per hospital visit based on literature [46]. In addition, time costs (i.e., those incurred due to absenteeism) were considered in both groups due to drug collection and/or routine follow-up, estimated at $49.97 per day based on the average monthly salary from the Chinese National Bureau of statistics in 2020 [21]. The time cost was involved in the Markov model until 55 years old, which is the mandatory age for retirement in China. The costs for each input variable are presented in Table 2. All costs in the Markov model were converted to US dollars based on an exchange rate of $1 = ¥6.4665 (as of 9 July 2021). In the model, a 5% annual discount rate was considered for all costs and benefits [47].

**Table 2:** Parameters input in the model and their ranges used in the sensitivity analyses

	Base-case values		Range			
	Capecitabine	Observation	Lower	Upper	Rule	Distribution^c^
Capecitabine for the first year ($/month)	306.48	0	26.84	306.48	Range^a^	Gamma
Monitoring safety of capecitabine therapy in the first year ($/month)	46.83	0	37.46	56.20	± 20%	Gamma
Treatment after relapse ($/month)^b^	1546.43	0	773.22	2319.65	± 50%	Gamma
Routine follow-up ($/month)						
< 3 years	38.39		30.71	46.07	± 20%	Gamma
3–5 years	21.32		17.06	25.58	± 20%	Gamma
> 5 years	12.78		10.22	15.34	± 20%	Gamma
Time cost ($/month)						
< 1 year	49.97	16.66	39.97 (capecitabine) 13.33 (observation)	59.96 (capecitabine) 19.99 (observation)	± 20%	Gamma
1–2 years	16.66		13.33	19.99	± 20%	Gamma
3–5 years	8.33		6.66	10.0	± 20%	Gamma
> 5 years	4.16		3.33	4.99	± 20%	Gamma
Travel cost ($/month)						
< 1 year	12.37	4.12	9.90 (capecitabine) 3.30 (observation)	14.84 (capecitabine) 4.94 (observation)	± 20%	Gamma
1–2 years	4.12		3.30	4.94	± 20%	Gamma
3–5 years	2.06		1.65	2.47	± 20%	Gamma
> 5 years	1.03		0.82	1.24	± 20%	Gamma
Management of grade 3/4 adverse events ($/case)						
Hand-foot syndrome	15.46	-	12.37	18.55	± 20%	Gamma
Diarrhea	44.30	-	28.50	54.60	[45]	Gamma
Incidence rate of grade 3/4 adverse events, %						
Hand-foot syndrome	13.45	0	11.44	15.47	95% CI	Beta
Diarrhea	3.19	0	2.15	4.22	95% CI	Beta
Utility						
DFS	0.80		0.73	0.87	[48]	Beta
Relapse	0.73		0.66	0.8	[48]	Beta
Disutility of grade 3/4 adverse events						
Hand-foot syndrome	0.12	-	0.096	0.144	± 10%	Beta
Diarrhea	0.10	-	0.08	0.12	± 10%	Beta
Annual discount rate, %	5		0	10	-	
Transition probability, %	Model fit		-	-	5%	Uniform

*-*, not applicable

^a^The range of capecitabine cost was set as the lowest and highest unit price from the Chinese Drug Bidding Database

^b^The monthly cost after relapse was estimated based on the monthly average treatment cost of relapsed cases in Sun Yat-sen University Cancer Center

^c^The distributions were applied in the probabilistic sensitivity analysis

*95% CI* 95% confidence interval, *DFS* Disease-free survival

### Utilities and outcome measures

Effectiveness was measured in quality-adjusted life years (QALYs), calculated as the survival time of one patient in a certain health state multiplied by the healthy utility value (quality of life weight) during the same period. According to a previous report [48], the utility values in the DFS, relapse, and death state were set to 0.8, 0.73, and 0 in this study, respectively. Disutility values due to grade 3/4 adverse events were set to 0.12 for hand-foot syndrome and 0.10 for diarrhea, respectively [49]. All adverse events were assumed to have been incurred in the first cycle [43].

The cost-effectiveness of capecitabine maintenance therapy versus routine follow-up was assessed by the incremental cost-effectiveness ratio (ICER), which is expressed as the incremental cost between two groups per QALY gained. Capecitabine maintenance therapy was considered cost-effective if the ICER was less than a willingness-to-pay (WTP) of $28,130 per QALY gained [50], which was three times that of China’s per-capita gross domestic product (GDP) according to the WHO guideline [20, 51].

### Sensitivity analyses

One-way and probabilistic sensitivity analyses were performed to evaluate the uncertainty of model parameters on ICER. In one-way sensitivity analysis, the parameters varied once at a time by their ranges specified in Table 2. A tornado chart was present to rank-order the parameters based on their potential impact on ICER. In probabilistic sensitivity analysis (PSA), a Monte Carlo simulation with 10,000 iterations was conducted by simultaneously sampling the model parameters from the distributions of each parameter. The sampling methods were set as gamma distribution for costs parameters, beta distribution for the incidence rate of severe adverse events, utility and disutility parameters, and uniform distribution for time-dependent state-transition probabilities parameters (Table 2). The PSA results were expressed as incremental cost-effectiveness scatter plots and cost-effectiveness acceptability curves.

### Calculation of number needed to treat

The number needed to treat (NNT) to prevent one additional relapse or death at a specific time point was calculated by fitting Cox proportional hazard model using the individual-level data of the SYSUCC-001 trial [52, 53], after adjustment of age, menopausal status, tumor size, node status, Ki67 index, surgery type, and treatment group. The NNT could be used to evaluate the treatment benefits of certain therapy in an absolute manner. A lower NNT indicated a higher clinical impact as fewer patients received the treatment without deriving a survival benefit from it. The costs to prevent one additional relapse were calculated based on the cost differences between the capecitabine maintenance and observational group.

## Supplementary Information

**Additional file 1: Table S1.** CHEERS 2022 Checklist. **Table S2.** Parameters used to fit survival curves in the five parametric models. **Table S3.** The results of goodness-of-fit to the individual-level data from the SYSUCC-001 trial. **Figure S1.** The fitted survival curves by five parametric distributions for the capecitabine maintenance and observational groups. **Table S4.** Time-dependent transition probabilities matrix of two groups. **Table S5.** The cost-effectiveness of capecitabine maintenance therapy based on alternative survival functions.

## Data Availability

The datasets used and/or analyzed during the current study are available from the corresponding author on reasonable request.

## Abbreviations

DFS: Disease-free survival
GDP: Gross domestic product
ICER: Incremental cost-effectiveness ratio
NNT: Number needed to treat
OS: Overall survival
PSA: Probabilistic sensitivity analysis
QALYs: Quality-adjusted life years
TNBC: Early-stage triple-negative breast cancer
WTP: Willingness-to-pay
