# Supplementary material for: A cost-effectiveness analysis of capecitabine maintenance therapy versus routine follow-up for early-stage triple-negative breast cancer patients after standard treatment from a perspective of Chinese society
Source: BMC Med. 2022 Sep 26;20:320. doi: 10.1186/s12916-022-02516-9 (PMC9511760; doi:10.1186/s12916-022-02516-9)
Supplement: Supplementary file 1 — Additional file 1: Table S1. CHEERS 2022 Checklist. Table S2. Parameters used to fit survival curves in the five parametric models. Table S3. The results of goodness-of-fit to the individual-level data from the SYSUCC-001 trial. Figure S1. The fitted survival curves by five parametric distributions for the capecitabine maintenance and observational groups. Table S4. Time-dependent transition probabilities matrix of two groups. Table S5. The cost-effectiveness of capecitabine maintenance therapy based on alternative survival functions. [file 12916_2022_2516_MOESM1_ESM.pdf]

Supplementary materials

Table S1. CHEERS 2022 Checklist

Topic	No.	Item	Reported
**Title**			
	1	Identify the study as an economic evaluation and specify the interventions being compared.	Yes
**Abstract**			
	2	Provide a structured summary that highlights context, key methods, results, and alternative analyses.	Yes
**Introduction**			
**Background and objectives**	3	Give the context for the study, the study question, and its practical relevance for decision making in policy or practice.	Yes
**Methods**			
**Health economic analysis plan**	4	Indicate whether a health economic analysis plan was developed and where available.	Yes
**Study population**	5	Describe characteristics of the study population (such as age range, demographics, socioeconomic, or clinical characteristics).	Yes
**Setting and location**	6	Provide relevant contextual information that may influence findings.	Yes
**Comparators**	7	Describe the interventions or strategies being compared and why chosen.	Yes
**Perspective**	8	State the perspective(s) adopted by the study and why chosen.	Yes
**Time horizon**	9	State the time horizon for the study and why appropriate.	Yes
**Discount rate**	10	Report the discount rate(s) and reason chosen.	Yes
**Selection of outcomes**	11	Describe what outcomes were used as the measure(s) of benefit(s) and harm(s).	Yes
**Measurement of outcomes**	12	Describe how outcomes used to capture benefit(s) and harm(s) were measured.	Yes
**Valuation of outcomes**	13	Describe the population and methods used to measure and value outcomes.	Yes
**Measurement and valuation of resources and costs**	14	Describe how costs were valued.	Yes
**Currency, price date, and conversion**	15	Report the dates of the estimated resource quantities and unit costs, plus the currency and year of conversion.	Yes
**Rationale and description of model**	16	If modelling is used, describe in detail and why used. Report if the model is publicly available and where it can be accessed.	Yes
**Analytics and assumptions**	17	Describe any methods for analysing or statistically transforming data, any extrapolation methods, and approaches for validating any model used.	Yes
**Characterising heterogeneity**	18	Describe any methods used for estimating how the results of the study vary for subgroups.	NA

Topic	No.	Item	Reported
**Characterising distributional effects**	19	Describe how impacts are distributed across different individuals or adjustments made to reflect priority populations.	No
**Characterising uncertainty**	20	Describe methods to characterise any sources of uncertainty in the analysis.	Yes
**Approach to engagement with patients and others affected by the study**	21	Describe any approaches to engage patients or service recipients, the general public, communities, or stakeholders (such as clinicians or payers) in the design of the study.	NA
**Results**			
**Study parameters**	22	Report all analytic inputs (such as values, ranges, references) including uncertainty or distributional assumptions.	Yes
**Summary of main results**	23	Report the mean values for the main categories of costs and outcomes of interest and summarise them in the most appropriate overall measure.	Yes
**Effect of uncertainty**	24	Describe how uncertainty about analytic judgments, inputs, or projections affect findings. Report the effect of choice of discount rate and time horizon, if applicable.	Yes
**Effect of engagement with patients and others affected by the study**	25	Report on any difference patient/service recipient, general public, community, or stakeholder involvement made to the approach or findings of the study	NA
**Discussion**			
**Study findings, limitations, generalisability, and current knowledge**	26	Report key findings, limitations, ethical or equity considerations not captured, and how these could affect patients, policy, or practice.	Yes
**Other relevant information**			
**Source of funding**	27	Describe how the study was funded and any role of the funder in the identification, design, conduct, and reporting of the analysis	Yes
**Conflicts of interest**	28	Report authors conflicts of interest according to journal or International Committee of Medical Journal Editors requirements.	Yes

From: Husereau D, Drummond M, Augustovski F, et al. Consolidated Health Economic Evaluation Reporting Standards 2022 (CHEERS 2022) Explanation and Elaboration: A Report of the ISPOR CHEERS II Good Practices Task Force. Value Health 2022;25. doi:10.1016/j.jval.2021.10.008

Table S2. Parameters used to fit survival curves in the five parametric models

	Exponential	Weibull		Log-normal		Log-logistic		Gamma	
	λ	γ	λ	μ	σ	γ	λ	γ	λ
**Disease-free survival**									
Capecitabine	0.00315	0.701	646.964	6.400	0.908	0.750	472.924	0.689	0.00115
Observation	0.00505	0.790	274.565	5.437	0.753	0.868	194.567	0.778	0.00293
**Overall survival**									
Capecitabine	0.00254	0.983	405.775	6.074	0.627	1.037	335.195	1.008	0.00259
Observation	0.00342	1.086	258.655	5.555	0.505	1.165	210.047	1.133	0.00451

Table S3. The results of goodness-of-fit to the individual-level data from the SYSUCC-001 trial

	Exponential	Weibull	Log-normal	Log-logistic	Gamma
**Disease-free survival**					
Capecitabine					
AIC	515.714	511.689	**505.371**	510.195	512.509
BIC	519.113	518.486	**512.167**	516.992	519.306
SSE	0.122	0.057	**0.047**	0.049	0.063
–2 log-likelihood statistic	-256.857	-253.845	**-250.685**	-253.098	-254.255
Observation					
AIC	706.276	704.399	**698.656**	702.309	705.133
BIC	709.637	711.122	**705.379**	709.031	711.855
SSE	0.096	0.043	**0.025**	0.031	0.051
–2 log-likelihood statistic	-352.138	-350.200	**-347.328**	-349.154	-350.566
**Overall survival**					
Capecitabine					
AIC	448.359	450.349	**446.195**	449.53	450.358
BIC	**451.758**	457.146	452.991	456.326	457.154
SSE	0.021	0.019	**0.015**	0.017	0.021
–2 log-likelihood statistic	-223.180	-223.175	**-221.097**	-222.765	-223.179
Observation					
AIC	549.658	551.354	**547.725**	550.201	551.154
BIC	**553.019**	558.076	554.447	556.923	557.877
SSE	0.022	0.028	**0.018**	0.022	0.029
–2 log-likelihood statistic	-273.829	-273.677	**-271.862**	-273.100	-273.577

SYSUCC: Sun Yat-sen University Cancer Center; AIC: Akaike information criterion; BIC: Bayesian information criterion; SSE: Sum of the squared errors.

(A) Disease-free survival

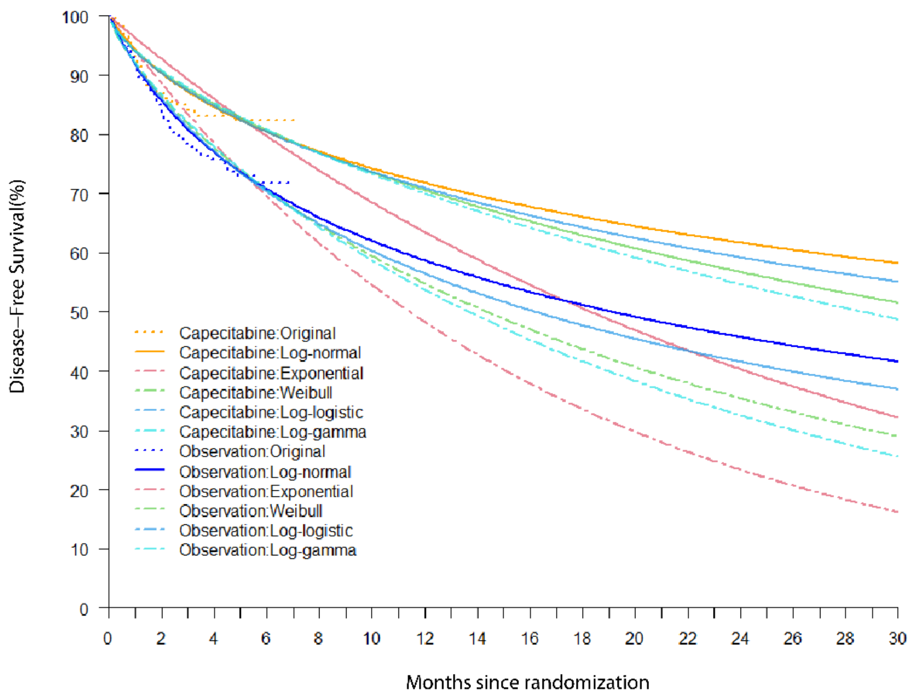

(B) Overall survival

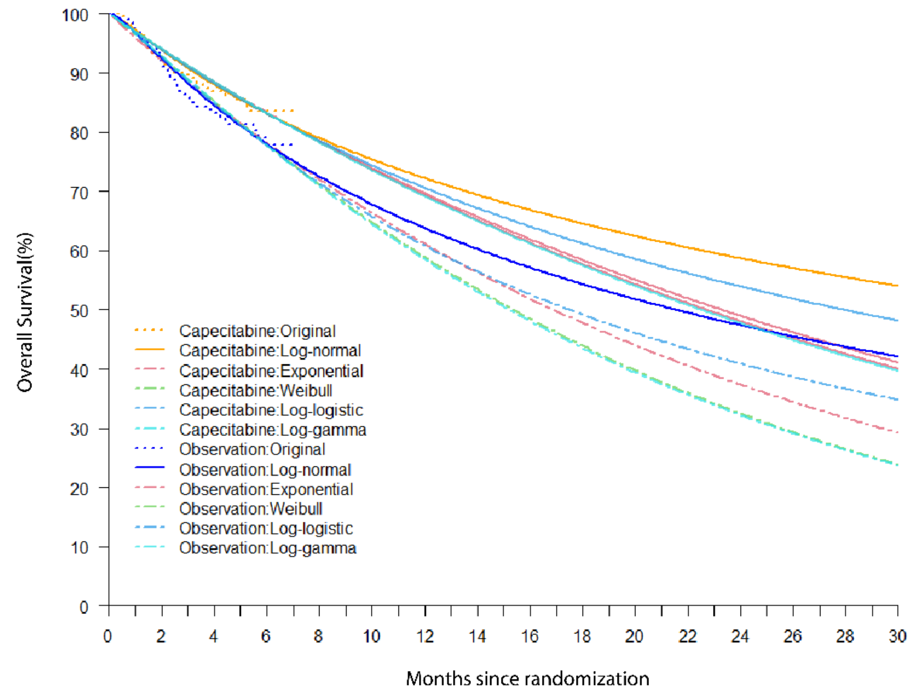

Figure S1. The fitted survival curves by five parametric distributions for the capecitabine maintenance and observational groups.

Table S4. Time-dependent transition probabilities matrix of two groups

	Cycle months	pFTF	pFTR	pFTD	pRTR	pRTD
Capecitabine maintenance group						
	1	0.995075	0.00483	9.50E-05	0.880343	0.119657
	2	0.994216	0.005689	9.50E-05	0.751205	0.248795
	3	0.994363	0.005542	9.50E-05	0.660326	0.339674
	4	0.994581	0.005324	9.50E-05	0.591029	0.408971
	5	0.994797	0.005108	9.50E-05	0.534812	0.465188
	6	0.994995	0.00491	9.50E-05	0.487484	0.512516
	7	0.995176	0.004729	9.50E-05	0.446635	0.553365
	8	0.99534	0.004565	9.50E-05	0.410735	0.589265
	9	0.995489	0.004416	9.50E-05	0.378745	0.621255
	10	0.995625	0.00428	9.50E-05	0.349927	0.650073
	11	0.99575	0.004155	9.50E-05	0.323736	0.676264
	12	0.995865	0.00404	9.50E-05	0.299756	0.700244
	……	……	……	……	……	……
	360	0.997784	0	0.00221598	0	1
Observational group						
	Cycle months	pFTF	pFTR	pFTD	pRTR	pRTD
	1	0.994786	0.005119	9.50E-05	0.923519	0.076481
	2	0.992458	0.007447	9.50E-05	0.831824	0.168176
	3	0.992126	0.007779	9.50E-05	0.758097	0.241903
	4	0.992146	0.007759	9.50E-05	0.699184	0.300816
	5	0.992276	0.007629	9.50E-05	0.650101	0.349899
	6	0.992445	0.00746	9.50E-05	0.608046	0.391954
	7	0.992625	0.00728	9.50E-05	0.571288	0.428712
	8	0.992805	0.0071	9.50E-05	0.538677	0.461323
	9	0.99298	0.006925	9.50E-05	0.509407	0.490593
	10	0.993148	0.006757	9.50E-05	0.482887	0.517113
	11	0.993307	0.006598	9.50E-05	0.458672	0.541328
	12	0.993459	0.006446	9.50E-05	0.436416	0.563584
	……	……	……	……	……	……
	360	0.997784	0	0.00221598	0	1

pFTF: Transition probability from disease-free state to disease-free state.

pFTR: Transition probability from disease-free state to relapse state.

pFTD: Transition probability from disease-free state to death state.

pRTR: Transition probability from relapse state to relapse state.

pRTD: Transition probability from relapse to death state.

Table S5. The cost-effectiveness of capecitabine maintenance therapy based on alternative survival functions

Survival functions	Effectiveness (QALYs)	Incremental Effectiveness	Cost ($)	Incremental Cost	ICER ($ per QALY)
Exponential					
Observation	6.92	—	4176.94	—	—
Capecitabine	8.36	1.44	8327.07	4150.13	2876.78
Weibull					
Observation	7.55	—	5238.13	—	—
Capecitabine	9.12	1.57	8819.09	3580.96	2284.43
Log-logistic					
Observation	7.78	—	5218.24	—	—
Capecitabine	9.19	1.41	8825.03	3606.79	2561.02
Gamma					
Observation	7.43	—	5375.73	—	—
Capecitabine	9.04	1.61	8879.83	3504.10	2176.91

—: Not applicable.
ICER: Incremental cost-effectiveness ratio; QALY: Quality-adjusted life year.
